# Examination of Trace Metals and Their Potential Transplacental Transfer in Pregnancy

**DOI:** 10.3390/ijms23158078

**Published:** 2022-07-22

**Authors:** Jovana Jagodić, Slađan Pavlović, Slavica Borković-Mitić, Milan Perović, Željko Miković, Slađana Đurđić, Dragan Manojlović, Aleksandar Stojsavljević

**Affiliations:** 1 Faculty of Chemistry, University of Belgrade, Studentski trg 12-16, 11000 Belgrade, Serbia; jovanaj@chem.bg.ac.rs (J.J.); sladjanadj@chem.bg.ac.rs (S.Đ.); manojlo@chem.bg.ac.rs (D.M.); 2 Institute for Biological Research “Siniša Stanković”—National Institute of the Republic of Serbia, University of Belgrade, Bulevar Despota Stefana 142, 11060 Belgrade, Serbia; sladjan@ibiss.bg.ac.rs (S.P.); borkos@ibiss.bg.ac.rs (S.B.-M.); 3 Clinic for Gynecology and Obstetrics Narodni Front, Faculty of Medicine University of Belgrade, Kraljice Natalije 62, 11000 Belgrade, Serbia; perovicmilan@hotmail.com (M.P.); mikovic.zeljko@gakfront.org (Ž.M.); 4 Innovative Centre of the Faculty of Chemistry, University of Belgrade, Studentski trg 12-16, 11000 Belgrade, Serbia

**Keywords:** umbilical cord serum, maternal serum, placenta, toxic trace metals, essential trace metals

## Abstract

With the ever-growing concern for human health and wellbeing, the prenatal period of development requires special attention since fetuses can be exposed to various metals through the mother. Therefore, this study explored the status of selected toxic (*Pb, Cd, Ni, As, Pt, Ce, Rb, Sr, U*) and essential trace metals (*Mn, Co, Cu, Zn, Se*) in the umbilical cord (UC) sera, maternal sera, and placental tissue samples of 92 healthy women with normal pregnancies. A further aim focuses on the potential transplacental transfer of these trace metals. Based on the obtained levels of investigated elements in clinical samples, it was observed that all of the trace metals cross the placental barrier and reach the fetus. Furthermore, statistical analysis revealed significant differences in levels of toxic *Ni, As, Cd, U, Sr, Rb*, and essential *Mn, Cu,* and *Zn* between all three types of analyzed clinical samples. Correlation analysis highlighted *As* to be an element with levels that differed significantly between all tested samples. Principal component analysis (PCA) was used to enhance these findings. PCA demonstrated that *Cd, Mn, Zn, Rb, Ce, U*, and *Sr* were the most influential trace metals in distinguishing placenta from maternal and UC serum samples. *As, Co*, and *Cu* were responsible for the clustering of maternal serum samples, and PCA demonstrated that the Pt level in UC sera was responsible for the clustering of these samples. Overall, the findings of this study could contribute to a better understanding of transplacental transfer of these trace metals, and shed a light on overall levels of metal exposure in the population of healthy pregnant women and their fetuses.

## 1. Introduction

Toxic trace metals and their impact on human health have been a cause for concern in recent times. Despite vast research, their effects on metabolism remain unclear, especially at low-level exposure [1,2]; however, it is known that uptake of certain trace metals contributes to increased oxidative stress by the formation of reactive oxygen species (ROS) [3]. Nevertheless, some health consequences of elevated toxic trace metal exposure during pregnancy have been recognized, including low fetal birth weight, preterm birth, and cognitive impairments, among others [4,5]. On the other hand, deleterious health impacts of essential trace metals receive less attention, regardless of their immense engagement in the enzymatic redox activities [6]. Considering the importance of essential trace metals, their status should be monitored on a frequent basis during pregnancy, particularly in maternal blood [7,8].

One of the most prominent hypothesized pathways for trace metal toxicity is oxidative stress, which is described as a homeostatic imbalance between cellular oxidants and antioxidant availability that favors oxidation [9]. Oxidative stress influences many adverse birth outcomes, including preeclampsia, premature delivery, and intrauterine growth restriction [10]. Since most trace metals, including essential trace metals, are redox-active, they have the potential to boost ROS generation [11]. Furthermore, due to differences in various metabolic pathways between the fetus and the mother, the fetus is highly sensitive to trace metals, even at low levels of exposure that do not affect the mother [12]. As a result, there was a greater focus on the impact of toxic trace metals on pregnancy and/or poor developmental outcomes, at levels lower than those prescribed in existing international regulations for blood, in order to elucidate the low-level exposure health effects [13].

Clinical samples, such as maternal sera/plasma are typically used in the majority of studies to evaluate the status of some redox-active trace metals in pregnancy, while umbilical cord (UC) sera/plasma and placental samples are used less frequently for this purpose [14]. Together, the analysis of these three types of clinical samples provides a plethora of information on trace metal exposure of both mother and the fetus. The placenta is the largest fetal organ, present for a limited period of time, and plays an important role in protecting the fetus from many pollutants [15,16]. In addition to being retrieved in a potentially non-invasive manner, the placenta reflects the fetal exposure to trace metals during pregnancy, making it an excellent sample for human exposure studies [17,18]. Although maternal whole blood, serum, and/or plasma are the commonly used types of clinical samples for trace metal dose/exposure assessment, placental tissue can provide important long-term information (9 months) on the levels of various metals that could affect the health of both the mother and fetus [17]. Furthermore, examining UC serum/plasma samples is a great means of detecting the elemental input of the fetus via the placenta and amniotic fluid [15]. UC serum can provide meaningful information about prenatal exposure in addition to being an easily assessed type of sample after the delivery [19].

Analyzing UC serum/plasma, maternal serum/plasma, and placental tissue samples is imperative for elemental profiling, because only by assessing all three types of samples can a comprehensive impression be obtained. Since fetuses can be exposed to many trace metals through their mothers, pregnancy requires particular consideration. Therefore, the following aims were addressed: to determine the status of selected toxic (*Pb, Cd, Ni, As, Pt, Ce, Rb, Sr, U*), and essential trace metals (*Mn, Cu, Zn, Se*) in a total of 276 samples (3 matrices × 92 participants) of UC serum, maternal serum, and placental tissue from healthy women with the normal course of pregnancies, and to indicate the potential transplacental transfer of trace metals.

## 2. Results

Table 1 shows the median, interquartile range (IQR), minimum (min), and maximum (max) levels of quantified essential and toxic trace metals in UC serum, maternal serum and placental samples, while Table S1 (Supplementary materials) shows the results of the Mann–Whitney U tests. Table 2 presents the results of Spearman’s rho (ρ) correlation analysis.

**Figure 1:**
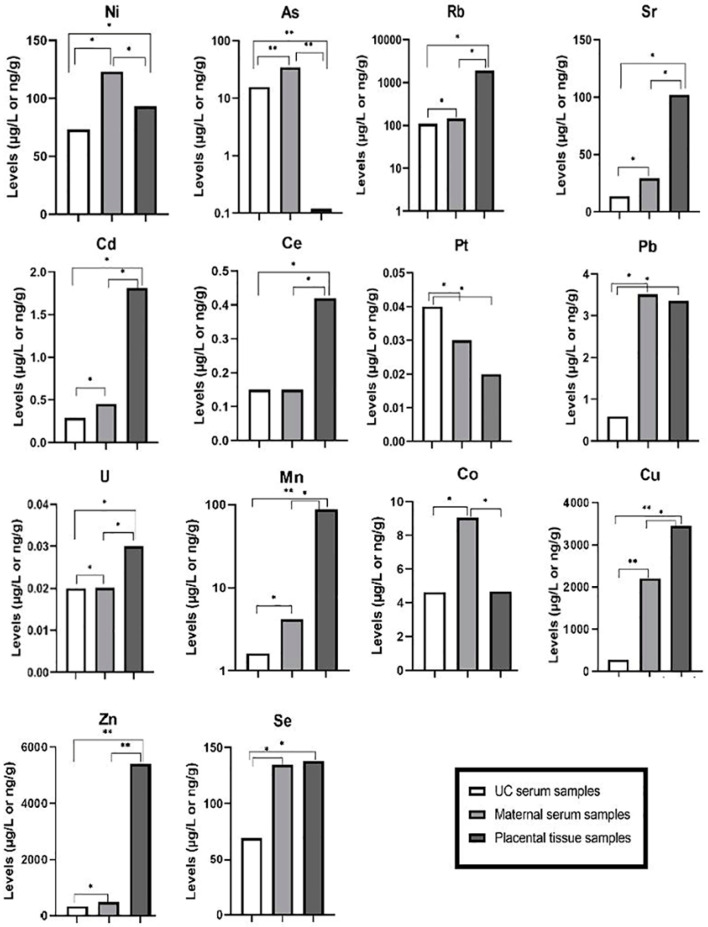
Levels of determined toxic and essential trace metals in UC serum (µg/L), maternal serum (µg/L) and placental tissue samples (ng/g). * *p* < 0.05; ** *p* < 0.01.

The median distribution of analyzed toxic trace metals in the UC sera were as follows: *Rb > Ni > As > Sr > Pb > Cd > Ce > Pt > U* (Table 1). Maternal serum samples had a similar distribution, except for Pt and U. Placental tissue samples showed different abundances: *Rb > Sr > Ni > Pb > Cd > Ce > As > U > Pt*. Regarding essential trace metals, the abundances were similar for all three types of clinical samples. The difference in the level for each examined trace metal in all three types of clinical samples is presented in Figure 1, together with the exact *p*-values obtained by the Mann–Whitney U test (Table S1). Thus, *Ni, As, Cd, U, Sr,* and *Rb* levels were significantly different between all tested samples. *Pb* and *Pt* did not significantly differ in placental and maternal serum samples, whilst *Ce* did not differ in UC and maternal sera. Considering essential trace metals, the *Mn, Cu*, and *Zn* were significantly different in all three types of clinical samples. *Co* did not show statistical significance only between UC serum and placental samples, whereas *Se* did not show significance only between maternal serum and placental samples (Figure 1 and Table S1).

Correlation analysis (Table 2) revealed that among all the trace elements, only *As* levels significantly differed between all types of samples; it showed a positive correlation between UC sera and placental tissue samples (0.287), but negative correlations between UC and maternal sera (−0.207), and placental tissue and maternal serum samples (−0.1998). Sr and Pt levels were different in maternal and UC sera. *Ni* was different in placental and maternal serum samples, while *Ce* was significantly different in placental samples and UC sera. *Cd, Pb, U*, and *Rb* did not show any significant correlations. From essential trace metals, correlation analysis singled out *Zn* in UC and maternal sera as being statistically significantly different.

**Figure 2:**
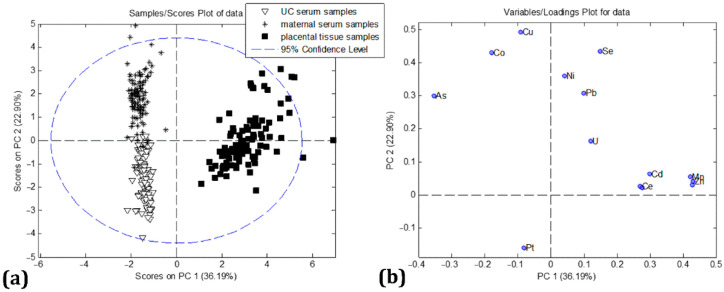
PCA model: (**a**) the score plot, (**b**) the loading plot for analyzed trace metals in maternal serum, placental tissue, and UC serum samples.

In order to gain a basic insight into the unique patterns of grouping among the studied samples, a multivariate principal component analysis (PCA) was performed. The four-component model of PCA explained 73% of total variance. The first principal component (PC1) covers 36.19% of the overall variance, while the second principal component (PC2) accounted for 22.87%. Figure 2 shows mutual projections of factor scores and their loadings for the PC1 and PC2. The score plot (Figure 2a) revealed three distinct groups of samples, representing placental samples, maternal and UC sera. Placental samples were clearly separated in the PC1 direction from maternal and UC samples. On the other hand, segmentation between the maternal and UC sera was observed in the PC2 direction. Furthermore, the loading plot (Figure 2b) revealed that the most influential trace metals that discriminated placental tissue from maternal and UC sera were *Cd, Mn, Zn, Rb, Ce, U,* and *Sr*; these trace metals showed the highest positive impact in the PC1 direction. The elements *As, Co,* and *Cu* were responsible for the clustering of maternal serum samples, showing a significant negative impact in the direction of PC1, i.e., a positive impact in the PC2 direction. On the other hand, the loading plot (Figure 2b) showed that the dominant *Pt* content in the UC sera was responsible for the grouping of these samples. The level of *Pt* was three times higher, on average, in the UC sera than in the other samples, resulting in this loading pattern. Trace metals *Ni, Pb*, and *Se* were close to zero in the PC1 direction, indicating a similar distribution of these trace metals in placental and maternal serum samples.

## 3. Discussion

### 3.1. Toxic Trace Metals

*Ni* is a toxic trace metal that plays a role in the inhibition of DNA repair, and the onset of numerous health impairments, including cardiovascular, renal, reproductive organ diseases, lung fibrosis, and cancer [20,21]. Once ingested, whether by food, air, or water, the absorption and cell entry pathway are determined by the chemical form of *Ni*; it has been suggested that soluble forms of *Ni* pass through cell membranes using *Ca* channels or by diffusion [20]. The toxicity of *Ni* could also be observed by its interference with the physiological processes of *Mn, Ca, Zn*, and *Mg* [22]. Furthermore, since *Ni* has embryotoxic and cytotoxic effects, it is necessary to monitor its levels in pregnant women [23]. We found *Ni* in UC serum, strongly suggesting that it can cross the placental barrier and reach the fetus. Nonetheless, the highest levels of *Ni* were observed in maternal serum, indicating that the placenta does serve as a semi-effective barrier to this toxic trace metal. Statistical analysis revealed a negative correlation between *Ni* levels in placental tissue and maternal serum, which supports the previously stated hypothesis.

*As* can be found in different oxidation states, as well as in organic and inorganic forms; it acts as an intermediate in metabolic reactions, and both methylated and dimethylated arsenicals containing *As* in the trivalent oxidation state have been found. Trivalent forms of *As* are more genotoxic and cytotoxic than pentavalent ones [24,25]. Many recent investigations have produced experimental confirmation that *As*-induced free radical production could induce DNA damage, and cell damage and death by activating oxidative signaling pathways [20,26]. In our study, the highest level of *As* was found in maternal sera, followed by UC sera, which suggests that the placenta could act as a semi-effective barrier to this toxic metal. Interestingly, of all the studied trace elements, *As* was the only one that proved significant in correlation analysis between all three types of clinical samples. *As* exposure in prenatal life increases the chance of detrimental health consequences, and has been linked to an increased risk of respiratory illness, cardiovascular disease, and cancer later in life [27]. Since fetuses are more susceptible to contaminants, maternal exposure to this toxic metal is of particular concern [28].

*Cd*, along with other toxic metals, can accumulate in different organs, such as the liver, lungs, kidneys, and testes; it has been reported that *Cd* can cause damage to gene expression by influencing the production of free radicals; mechanisms are by increasing lipid peroxidation and/or by changing intracellular glutathione levels [29]. Studies across the world have reported higher *Cd* levels in the placenta than in maternal plasma/serum and UC plasma/serum [13,16], which are in agreement with our results. The measured *Cd* levels in our investigated clinical samples could be explained by the limited transplacental passage of this toxic metal. Our current results also suggest that *Cd* accumulates in the placenta during pregnancy, providing credence to the hypothesis that this organ serves as an efficient or partially efficient barrier against *Cd* [30]. The placenta expresses metallothioneins, which could explain the accumulation of *Cd* in this organ [31].

*Pt* is considered a genotoxic and cytotoxic metal, and its toxicity greatly depends on the oxidation state and structure [32]. Among their uses in industry and catalysis processes, *Pt* compounds have found their roles in modern medicine as anticancer drugs, but they have a side effect of causing oxidative stress to the dorsal root ganglia [33]. Some studies suggested that *Pt* has a deleterious effect on the female reproductive system [34]. *Pt* levels were the highest in UC serum and lowest in the placental tissues in our study, strongly suggesting that there is no effective placental barrier to this metal. Furthermore, correlation analysis revealed a positive correlation in *Pt* levels between UC and maternal serum (Table 2), indicating that when *Pt* levels rise in maternal serum, they also rise in UC serum. The negative health effects of *Pt* on mothers and their fetuses are still not fully understood, necessitating further research.

Exposure to *Pb*, even at low levels, has been linked to a variety of health issues, including cognitive impairments, renal and cardiovascular problems, reproductive system impairments, and hypertension among others [35]. Furthermore, *Pb* can have many negative impacts on the pregnancy, including detrimental cognitive development, low birth weight, and premature labor [36]. Besides its potential to accumulate in some organs (such as bones), *Pb* could influence ROS generation. Ahamed and Siddiqui mentioned that *Pb* could have the capacity to stimulate ferrous ion-initiated membrane lipid peroxidation [35,37]. Usually, antioxidant molecules such as glutathione and glutathione disulfide levels, and activities of antioxidant enzymes, superoxide dismutase, catalase, glutathione peroxidase, and glutathione reductase, are the most commonly used parameters to evaluate *Pb*-induced oxidative damage [37]. Iwai-Shimada et al. mentioned the possibility that *Pb* ions could mimic *Ca* ions [13]. Levels of *Pb* in UC serum were significantly lower than those of maternal serum and placental tissue in our study. The presence of higher *Pb* in our placental samples than in UC serum indicates that the placenta acts as a potential barrier and possibly accumulates *Pb* during pregnancy. Furthermore, the detectability of *Pb* in UC serum suggests that this toxic metal passes through the placental barrier. Additional research is needed to investigate the nature of *Pb* transplacental transfer, as it is questionable whether the transfer occurs actively or passively.

### 3.2. Essential Trace Metals

Since *Mn* is an important part of catalase and *Mn*-containing superoxide dismutase (MnSOD), it has a role in minimizing oxidative stress through the detoxification of superoxide-derived free radicals. *Mn* is essential for fetal development, and inadequate levels—both high and low—can cause detrimental birth outcomes [38]. For example, inadequate UC serum *Mn* levels during pregnancy could be associated with impaired fetal growth, bone formation, immune function, and neurodevelopment [39,40]. Studies have shown that *Mn* levels tend to rise in the mother’s blood during pregnancy in order to obtain sufficient levels for fetal growth and development [39,40,41]. Our results show that placental tissue had the most abundant *Mn* levels, followed by maternal serum; it has been suggested in the literature that *Mn* crosses the placenta via active transport [42].

*Co* has many important roles in metabolism, including in amino acid and nucleic acid synthesis as well as in the formation of erythrocytes; it also has an impact on oxidative stress and inflammatory responses, by regulating inflammatory cytokines as a part of vitamin B12 [43,44]. Furthermore, if *Co* is deficient during pregnancy, vitamin B12 production will be disrupted, which raises the likelihood of developmental defects in newborns [45]. In our study, the maternal serum had the highest levels of *Co*, followed by UC serum. Interestingly, UC serum and placental tissue had similar *Co* levels. Our finding indicates that transplacental transfer of *Co* must be tightly regulated, and further studies are needed to reveal this unresolved mechanism.

*Cu*, as a metal with high redox potential, acts as a cofactor for different proteins involved in numerous reactions, such as iron metabolism, free radical elimination, respiration, connective tissue generation, and normal neurological function and its physiological levels in the body are vital for normal cell function [20]. *Cu* is necessary for the process of neural myelination; it is also involved in the metabolism of neurotransmitters and in the regulation of other metals in the body, primarily *Zn. Cu*, along with *Zn*, is an integral part of the active center in *CuZn*-containing superoxide dismutase [46]. Clinical *Cu* deficiency, resulting from a lack maternal dietary intake, is an extremely rare occurrence; however, mild *Cu* deficiency mediated by secondary reasons (illness, pharmaceuticals, nutritional or genetic factors, etc.) is more prevalent and can lead to concerns during pregnancy [47]. Kot et al. pointed out those low *Cu* levels have been linked to an increased risk of giving birth to low-birth-weight neonates [3]. Higher levels of maternal *Cu* are an indicator of estrogen-induced release of *Cu* from storage in the mother’s body during pregnancy, suggesting an increased requirement for this microelement at this time [3,47]. Furthermore, due to estrogen-induced changes, reference values for *Cu* levels in pregnant women’s sera/plasma differ from those of nonpregnant women. *Cu* transfer from mother to fetus is tightly regulated by chaperone proteins [48]. Chaperon-like actions could explain our results of the highest *Cu* levels being in maternal serum, followed by placental tissue. UC serum had the lowest *Cu* levels compared to other types of clinical samples.

Among its many roles, *Zn*, an essential and redox-inactive trace metal, is pivotal for normal immune system function, bone and teeth mineralization, and management of blood glucose concentration. Inadequate intake of this essential metal can lead to various health concerns [20,47]. High phytate intake, chronic illness, or *Fe* or *Cu* oversupplementation can also be the causes for *Zn* deficiency [20,49]. Symptoms of *Zn* deficiency include poor growth and development, appetite loss, delayed wound healing, dermatitis, hypogonadism, impaired reproduction, and poor immune function. Furthermore, *Zn* deficiency has been linked to higher-than-normal levels of oxidative damage in tissues, including increased lipid, protein, and DNA oxidation [20,49]. Pregnant women from underdeveloped nations are more likely to have *Zn* deficiency, and several studies have suggested *Zn* supplementation as a preventive measure [47,50]. Furthermore, pregnant women have lower *Zn* levels than nonpregnant healthy women as a natural result of higher *Zn* absorption by the fetus and placenta, as well as the mother’s expanded plasma volume, hormonal changes, and increased urinary *Zn* excretion [51]. In our study, the highest levels of *Zn* were found in placental tissue, indicating the possible accumulation of this essential trace metal. Furthermore, correlation analysis revealed a negative correlation between maternal and UC serum, indicating that when *Zn* levels in UC serum increase, they decrease in maternal serum and vice versa.

*Se* has a major role in the activity of antioxidative enzymes and in antioxidant defense in mothers and their fetuses [52]. As a part of glutathione peroxidase, *Se* helps the prevention of adverse health effects in pregnancy, such as neural tube defects, miscarriages, and gestational diabetes [52,53]. The level of *Se* in maternal blood decreases markedly during pregnancy; moreover, alterations in *Se* homeostasis during this sensitive period are most likely caused by an increased demand for oxygen in both the mother’s and the developing fetus’s bodies [54]. Studies have shown that *Se* deficiency contributes to increased levels of miscarriage and impairments to the fetuses nervous and immune systems. A low level of *Se* in serum during the early stages of pregnancy has been shown to be a predictor of a newborn’s low birth weight [55]. Our findings show that *Se* levels in maternal serum and placental tissue are fairly similar and higher than in UC serum, indicating that the *Se* transport mechanism is tightly regulated, although the mechanism by which it is regulated is unknown. Although the correlation analysis showed no statistical significance with respect to *Se* levels, negative correlations were observed between *Se* levels in UC and maternal serum, and in UC serum and placental tissue, while maternal serum and placental tissue exhibited a positive correlation; these findings can possibly provide insight into the mechanism of what seems to be tightly regulated *Se* transport.

## 4. Materials and Methods

### 4.1. Collection of Samples

All women were interviewed face-to-face by a professional interviewer and all were informed of the goals and procedures of the study. Through a meticulous selection process, 92 women were enrolled and gave their written informed consent. Participating women were, on average, in the 40th gestational week and were from 20 to 35 years of age. The strictly followed inclusion criteria incorporated singleton pregnancies, without complicated deliveries. Exclusion criteria stated those women who were smokers, who were professionally exposed to trace elements, who had current use of drugs or supplements that could affect their trace metal levels, and who had abnormal vaginal bleeding, multiple pregnancies, gestational diabetes, or renal or liver diseases were to not be included in the research.

A whole blood specimen from each pregnant woman was obtained before delivery, by withdrawing 4.5 mL of blood from a median cubital vein from the anterior elbow fold with a silicone needle and disposable syringe, and dispensing it into a BD 368381 Vacutainer Trace Element Plastic Blood Collection Tube (BD Vacutainer^®^). From each participant after delivery, whole UC blood was collected from the cut and clamped umbilical cord, using a silicone needle and disposable syringe; this blood was also dispensed into a BD Vacutainer tube. Every whole blood specimen obtained was tested for indicators of infectious diseases (hepatitis B and C, toxoplasmosis, syphilis, HIV, CMV, and HTLV-1 and 2), and all were found to be negative. Following spontaneous coagulation, which lasted for about 30 min, whole blood samples were centrifuged for 10 min at 3000× *g*, and obtained sera were transferred to trace element-free Eppendorf tubes. Placental tissue samples (around 0.30 g) were taken after delivery from the lowest areas of the cord tissue root, and placed in trace element-free Eppendorf tubes. All collected sera and tissue samples were frozen at −80 °C and stored until analysis. 

### 4.2. Trace Element Analysis

UC and maternal serum samples were prepared by a 10-fold dilution with an aqueous solution containing nitric acid (0.05%), Triton X-100 (0.1%), and 1-butanol (3%). In order to aid the precision of our quantitative analysis, internal standard solution (^45^*Sc* at a concentration of 50 µg/L and ^71^*Ga,* ^115^*In,* ^159^*Tb* at concentrations of 10 µg/L) was added to every sample in equal amounts.

Placental samples were firstly precisely weighed on an analytical balance and then placed in microwave cuvettes, to which a mixture of nitric acid and hydrogen peroxide (4:1, *v*:*v*) was added; these samples were decomposed with microwave digestion, in an ETHOS 1 (Milestone, Italy) apparatus. The method that was utilized firstly gradually heated the microwave cuvettes for 4 min to 90 °C, and then heated for 6 min to 115 °C, then 8 min to 120 °C, which was maintained as the final temperature for 20 min. After finished microwave digestion of the placental samples, the cuvettes were left to cool and their contents were diluted with ultrapure water in 25 mL glass volumetric flasks.

All samples were analyzed in triplicate. Calibration curves exhibited linearity of R ≥ 0.997 in the range of 1 to 300 µg/L (*Cu, Zn, Se, Rb*), 1–30 µg/L (*Mn, Co, Ni, As, Sr, Cd, Pb*), and 0.1–30 µg/L (*Ce, Pt, U*). The investigated trace elements were quantified by the inductively coupled plasma quadrupole mass spectrometry (ICP-Q-MS) (iCAP Q*_c_*, Thermo Scientific, Oxford, UK) technique with helium mode. The accuracy of the utilized analytical technique was tested using certified reference materials (CRMs) manufactured by Seronorm^TM^ (Trace Elements Serum Level-1 and Level-2) and NIST (bovine liver 1577c), and the obtained recoveries ranged from 81.1 to 117%. The following isotopes of trace metals were chosen based on the CRM recoveries: ^55^*Mn,* ^59^*Co,* ^60^*Ni,* ^65^*Cu,* ^67^*Zn,* ^75^*As,* ^78/82^*Se,* ^87^*Rb,* ^88^*Sr,* ^111^*Cd,* ^142^*Ce,* ^196^*Pt,* ^208^*Pb*, and ^238^*U.*

### 4.3. Data Analysis

For data analysis, the statistical software SPSS (IBM SPSS Statistics, version 20) was utilized. The Kolmogorov-Smirnov test was used to check the normality of data. To detect possible variations in elemental levels between clinical sample types, the Mann–Whitney U test was utilized. Correlation analysis was conducted using Spearman’s rho (ρ) test. Spearman’s rho (ρ) test was chosen as a nonparametric test, which is in line with the normality of data; it is a commonly used statistical method that measures the strength and direction of association between two sets of data when ranked by each of their quantities. Principal component analysis (PCA) was performed by PLS ToolBox, v.6.2.1, for MATLAB v. 7.12.0 (R2011a); this multivariate method creates a new coordinate system consisting of mutually normal latent variables directed in the direction of the greatest variability among the data. The advantages of PCA are reflected in the clear definition of the method and the possibility of interpreting the results through score graphs and graphs of coefficients of latent variables compared to non-linear methods (cluster analysis, factor analysis). In addition, the disadvantage of non-linear methods in relation to PCA is the existence of parameters that can be adjusted (methods for determining the distance of objects and methods for connecting them in cluster analysis; data reduction in factor analysis) [56]. *p* < 0.05 was considered the cut-off value for all tests.

## 5. Conclusions

This study quantified selected toxic (*Pb, Cd, Ni, As, Pt, Ce, Rb, Sr, U*) and essential trace metals (*Mn, Co, Cu, Zn, Se*) in UC serum, maternal serum, and placental tissue from healthy women with normal pregnancies. Results revealed that all analyzed trace metals pass through the placental barrier and reach the fetus. Mann–Whitney U tests showed that there were statistically significant differences regarding levels of toxic *Ni, As, Cd, U, Sr, Rb*, and essential *Mn, Cu,* and *Se* in the analyzed samples. Correlation analysis singled out *As* levels, which significantly differed in all types of tissues. *Cd, Mn, Zn, Rb, Ce, U*, and *Sr* were identified as the most important trace metals in differentiating placenta from maternal and UC serum samples using PCA. Furthermore, PCA revealed that levels of *As, Co*, and *Cu* were accountable for the clustering of maternal serum samples, while the Pt levels were significant for the clustering of UC sera. The findings of this study could lead to a better understanding of transplacental metal transfer and overall exposures to these trace metals in a population of healthy pregnant women and their fetuses. Given the numerous short and long-term effects of toxic metal exposure and inadequate levels of essential metals on organogenesis, fetal health, pregnancy, and birth outcomes, a better understanding of the exact pathways of accumulation and transport in the human placenta is essential.

**Study limitations:** Despite the large number of samples of UC sera, maternal sera, and placental tissue samples from the same women being analyzed, there were no statistically significant findings for the metals studied when split by age groups. As a result, future studies should concentrate on gathering more samples from various age groups in order to undertake a comprehensive statistical analysis. Furthermore, pairwise comparisons for multiple samples were not performed in this study.

## Figures and Tables

**Table 1:** Median, interquartile range (IQR), and minimum (min) and maximum (max) levels of the toxic and essential metals in the analyzed clinical samples.

		Ni	As	Rb	Sr	Cd	Ce	Pt	Pb	U	Mn	Co	Cu	Zn	Se
**Umbilical cord serum** **(µg/L)**	Median	73.2	15.70	110.0	13.50	0.29	0.15	0.040	0.59	0.020	1.61	4.63	273.0	338.0	69.3
	IQR	38.3	11.40	85.2	8.73	0.20	0.11	0.050	0.50	0.020	1.04	5.99	151.0	180.0	32.1
	Min	21.3	2.51	20.7	3.92	0.01	0.01	0.003	0.01	0.003	0.12	1.21	56.1	95.3	23.4
	Max	184.0	39.60	295.0	41.10	0.77	0.39	0.720	4.14	0.090	4.34	8.69	634.0	755.0	146.0
**Maternal** **serum** **(µg/L)**	Median	123.0	34.60	144.0	29.20	0.45	0.15	0.030	3.51	0.020	4.21	9.05	2200.0	472.0	135.0
	IQR	19.4	8.01	39.7	11.30	0.24	0.13	0.030	5.29	0.030	1.02	1.31	507.0	92.9	35.9
	Min	74.8	16.90	63.6	15.40	0.04	0.01	0.003	0.11	0.010	1.69	5.42	547.0	180.0	55.1
	Max	168.0	60.80	246.0	69.30	2.65	0.67	0.080	19.20	0.310	9.23	12.10	3446.0	1420.0	247.0
**Placental** **tissue** **(ng/g)**	Median	93.3	0.12	1923.0	102.00	1.81	0.42	0.020	3.36	0.030	87.80	4.09	859.0	5401.0	138.0
	IQR	85.2	0.11	527.0	78.80	1.43	0.53	0.020	3.26	0.040	31.20	2.48	198.0	1440.0	32.2
	Min	14.6	0.02	664.0	28.80	0.33	0.02	0.001	0.13	0.001	49.40	1.36	401.0	2840.0	34.1
	Max	466.0	2.51	3306.0	650.00	16.70	5.76	0.090	31.10	0.280	237.00	20.40	1616.0	10171.0	188.0

**Table 2:** Results of the Spearman’s rho (ρ) correlation analysis between different types of clinical samples. Statistically significant values are given in bold.

		Ni	As	Rb	Sr	Cd	Ce	Pt
UC serum	Maternal serum	−0.1598	**−0.2070**	−0.1154	**−0.2494**	0.1158	−0.0427	**0.4162**
UC serum	Placental tissue	0.1375	**0.2872**	−0.1170	0.1735	0.1106	**−0.2208**	−0.0493
Maternal serum	Placental tissue	**−0.2244**	**−0.1998**	0.0083	−0.0289	−0.0227	−0.0348	0.0428
		**Pb**	**U**	**Mn**	**Co**	**Cu**	**Zn**	**Se**
UC serum	Maternal serum	0.0808	0.0423	−0.0414	0.1410	−0.1396	**−0.2182**	−0.1232
UC serum	Placental tissue	0.1284	−0.0807	−0.0675	0.1494	−0.1411	−0.0888	−0.0537
Maternal serum	Placental tissue	−0.0876	−0.0043	0.0011	−0.1374	0.0829	−0.0198	0.1056

## Data Availability

The data presented in this study are available on a reasonable request from the corresponding author.

## Supplementary Materials

The following supporting information can be downloaded at: https://www.mdpi.com/article/10.3390/ijms23158078/s1.
