# Supplementary material for: Examination of Trace Metals and Their Potential Transplacental Transfer in Pregnancy
Source: Int J Mol Sci. 2022 Jul 22;23(15):8078. doi: 10.3390/ijms23158078 (PMC9330144; doi:10.3390/ijms23158078)
Supplement: Supplementary file 1 [file ijms-23-08078-s001.zip › ijms-1825295-supplementary.pdf]

Supplementary material

Table S1. Results of the Mann-Whitney U test, where values below 0.05 were considered as statistically significant. Non-significant relationships are shown in *italics*.

		**Ni**	**As**	**Rb**	**Sr**	**Cd**	**Ce**	**Pt**	**Pb**	**U**	**Mn**	**Co**	**Cu**	**Zn**	**Se**
UC serum	Maternal serum	0.015	<0.01	0.041	0.035	0.036	*0.670*	0.039	0.018	0.047	0.029	0.031	<0.01	0.042	0.039
UC serum	Placental tissue	0.024	<0.01	0.032	0.022	0.017	0.025	0.035	021	0.036	<0.01	*0.542*	<0.01	<0.01	0.037
Maternal serum	Placental tissue	0.031	<0.01	0.034	0.022	0.019	0.027	*0.071*	*0.419*	0.037	0.014	0.032	0.033	<0.01	*0.342*
